# Supplementary material for: Metabolic engineering of Escherichia coli for optimized biosynthesis of nicotinamide mononucleotide, a noncanonical redox cofactor
Source: Microb Cell Fact. 2020 Jul 27;19:150. doi: 10.1186/s12934-020-01415-z (PMC7384224; doi:10.1186/s12934-020-01415-z)
Supplement: Supplementary file 1 — Additional file 1: Figure S1.Francisella tularensis NadE*-based Growth Restoration is Not Nicotinamide Feeding Dependent. A growth restoration platform was used to screen pathways for the efficient generation of nicotinamide mononucleotide (NMN+). The Escherichia coli strain 72c [1], note this reference number is for additional files only] contains a temperature sensitive allele of nadD (ts nadD). As a result, this strain cannot grow at 42 °C. By overexpressing Francisella tularensis nadE*, cells are able to produce NMN+, which can then be converted to NAD+, and thus restoring growth. We observed no dependence of growth restoration with feeding 200 µM nicotinamide (NA). This indicates either efficient NMN+ generation can be achieved through channeling the intermediate nicotinic acid mononucleotide (NaMN+) from E. coli ‘s native de novo NAD+ biosynthesis pathway, or LB medium used in this experiment already contains sufficient precursors for this pathway. Screening was performed in a deep-well 96-well plate containing 1 mL of LB medium supplemented with 2 g/L d-glucose and 200 μM of NA if applicable. Detailed conditions are described in the Methods section. Figure S2.Francisella tularensis NadE* Expression Alleviates R. solanacearum NadV Growth Challenge. BW25113 ΔpncC cells expressing R. solanacearum NadV demonstrate a growth challenge. However, when paired with F. tularensis NadE*, the growth challenge is alleviated. Interestingly, this challenge is not seen when H. ducreyi NadV is expressed. Therefore, F. tularensis NadE* may play a synergistic role in stability, activity, or expression of some NadV candidates. Cells were cultured identically to the intracellular NMN+ generation cultures described in the Methods section. Cell growth was monitored by measuring optical density at 600 nm. Abbreviations indicate source of genes: Ft, Francisella tularensis, Hd, Haemophilus ducreyi; Rs, Ralstonia solanacearum. Figure S3. Intracellular NAD+ Decreases in NMN+ Accumulating [file 12934_2020_1415_MOESM1_ESM.pdf]

Metabolic engineering of *Escherichia coli* for optimized biosynthesis of nicotinamide mononucleotide, a noncanonical redox cofactor

William B. Black [1], Derek Aspacio [1], Danielle Bever [1], Edward King [2], Linyue Zhang [1], and Han Li [1,*]

Departments of Chemical and Biomolecular Engineering[1], Molecular Biology and Biochemistry[2], University of California, Irvine. *To whom correspondence should be addressed

Supplementary information

Supplementary Figure 1: *Francisella tularensis* NadE*-based Growth Restoration is Not Nicotinamide Feeding Dependent.

Supplementary Figure 2: *Francisella tularensis* NadE* Expression Alleviates *R. solanacearum* NadV Growth Challenge

Supplementary Figure 3: Intracellular NAD^+ Decreases in NMN^+ Accumulating Strains.

DNA sequences of genes used in this study

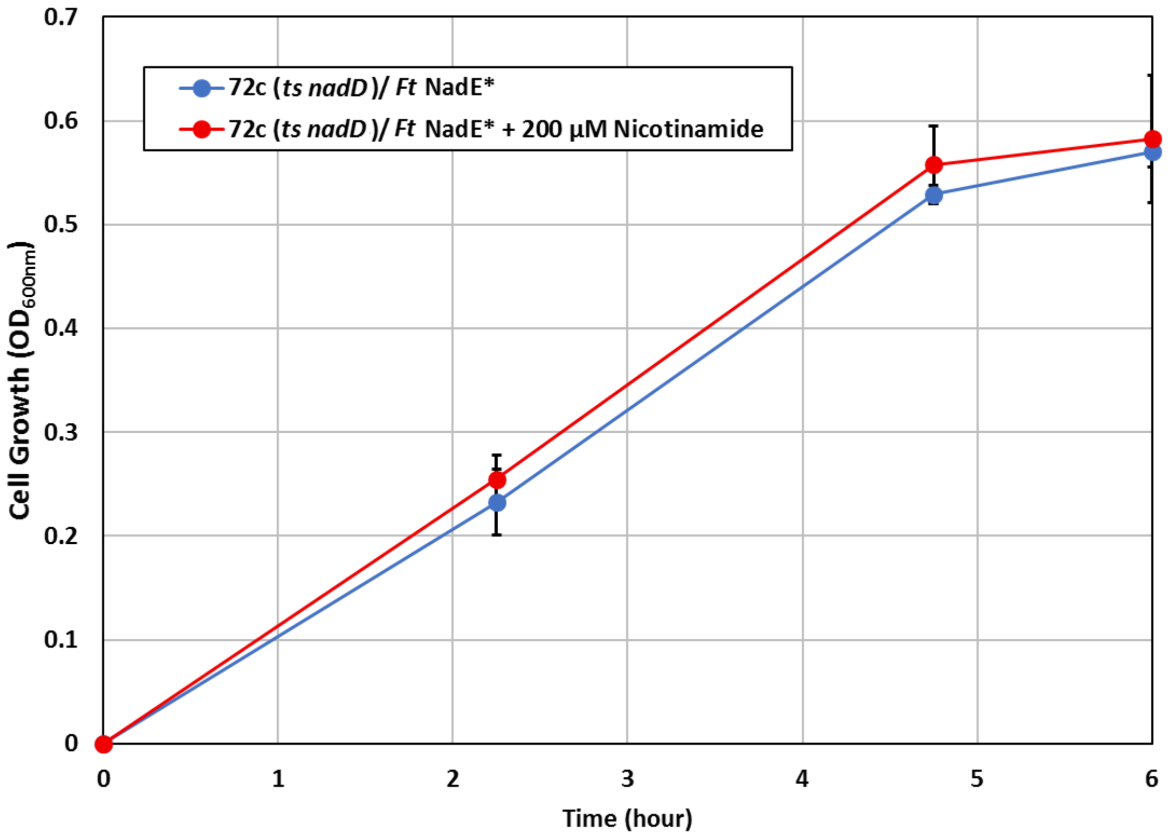

Supplementary Figure 1: *Francisella tularensis* NadE*-based Growth Restoration is Not Nicotinamide Feeding Dependent

A growth restoration platform was used to screen pathways for the efficient generation of nicotinamide mononucleotide (NMN^+). The *Escherichia coli* strain 72c [1] contains a temperature sensitive allele of *nadD* (*ts nadD*). As a result, this strain cannot grow at 42 ℃. By overexpressing *Francisella tularensis nadE**, cells are able to produce NMN^+, which can then be converted to NAD^+, and thus restoring growth. We observed no dependence of growth restoration with feeding 200 µM nicotinamide (NA). This indicates either efficient NMN^+ generation can be achieved through channeling the intermediate nicotinic acid mononucleotide ($NaMN^+$) from *E. coli* 's native *de novo* NAD^+ biosynthesis pathway, or LB medium used in this experiment already contains sufficient precursors for this pathway. Screening was performed in a deep-well 96-well plate containing 1 mL of LB medium supplemented with 2 g/L D-glucose and 200 µM of NA if applicable. Detailed conditions are described in the Methods section.

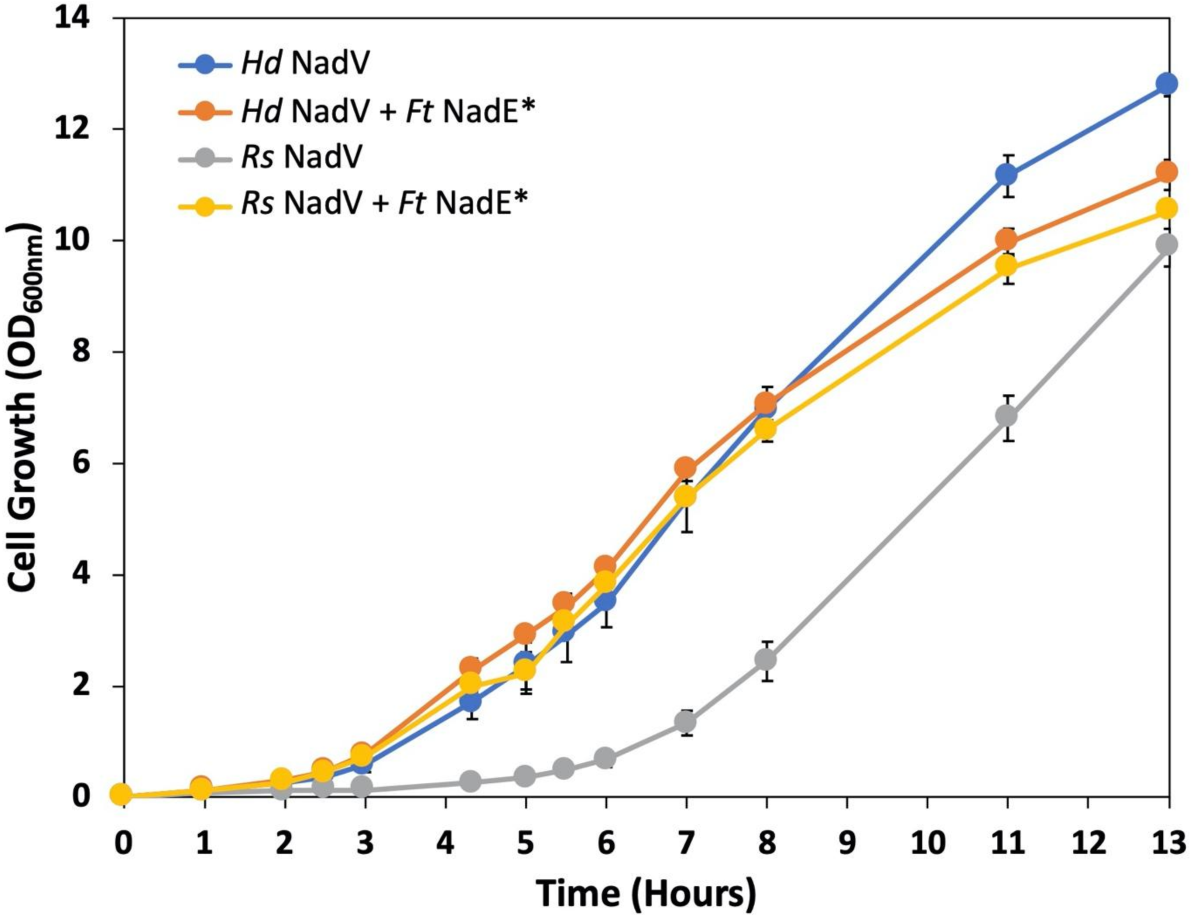

Supplementary Figure 2: *Francisella tularensis* NadE* Expression Alleviates *R. solanacearum* NadV Growth Challenge

BW25113 ∆*pncc* cells expressing *R. solanacearum* NadV demonstrate a growth challenge. However, when paired with *F. tularensis* NadE*, the growth challenge is alleviated. Interestingly, this challenge is not seen when *H. ducreyi* NadV is expressed. Therefore, *F. tularensis* NadE* may play a synergistic role in stability, activity, or expression of some NadV candidates. Cells were cultured identically to the intracellular NMN^+ generation cultures described in the Methods section. Cell growth was monitored by measuring optical density at 600 nm. Abbreviations indicate source of genes: *Ft*, *Francisella tularensis*, *Hd*, *Haemophilus ducreyi*; *Rs*, *Ralstonia solanaceaum*.

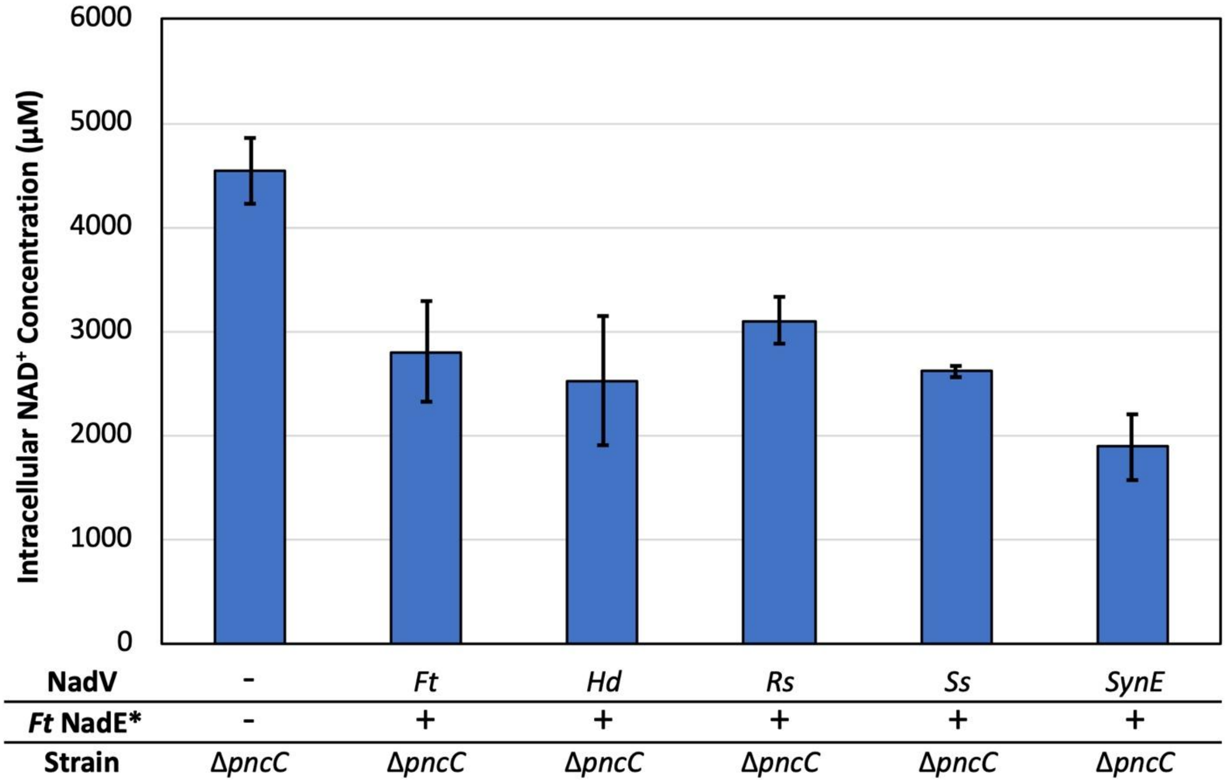

Supplementary Figure 3: Intracellular NAD^+ Decreases in NMN^+ Accumulating Strains
From Figure 3, co-overexpression of NMN^+ generating *Francisella tularensis* NadE* and NadVs increases intracellular NMN^+ when the NMN^+ degrading PncC is disrupted. However, as shown here, NAD^+ levels decreased in cells expressing *F. tularensis* NadE* and NadV compared to cells without overexpression. This potentially indicates NMN^+ plays a regulatory role in NAD^+ biosynthesis. Cells were cultured in 2xYT medium supplemented with 1 mM nicotinamide at 30 ℃ for four hours. Intracellular NAD^+ concentrations were determined by UPLC-MS/MS. Detailed conditions and analytical techniques are described in the Methods section.

DNA sequences of genes used in this study

Escherichia coli* BL21 *yqhD

ATGAACAACTTTAATCTGCACACCCCAACCCGCATTCTGTTTGGTAAAGGCGCAATC
GCTGGTTTACGCGAACAAATTCCTCACGATGCTCGCGTATTGATTACCTACGGCGGC
GGCAGCGTGAAAAAAACCGGCGTTCTCGATCAAGTTCTGGATGCCCTGAAAGGCAT
GGACGTGCTGGAATTTGGCGGTATTGAGCCAAACCCGGCTTATGAAACGCTGATGA
ACGCCGTGAAACTGGTTCGCGAACAGAAAGTGACTTTCCTGCTGGCGGTTGGCGGC
GGTTCTGTACTGGACGGCACCAAATTTATCGCCGCAGCGGCTAACTATCCGGAAAAT
ATCGATCCGTGGCACATTCTGCAAACGGGCGGTAAAGAGATTAAAAGCGCCATCCC
GATGGGCTGTGTGCTGACGCTGCCAGCAACCGGTTCAGAATCCAACGCAGGCGCGG
TGATCTCCCGTAAAACCACAGGCGACAAGCAGGCGTTCCATTCTGCCCATGTTCAGC
CGGTATTTGCCGTGCTCGATCCGGTTTATACCTACACCCTGCCGCCGCGTCAGGTGG
CTAACGGCGTAGTGGACGCCTTTGTACACACCGTGGAACAGTATGTTACCAAACCGG
TTGATGCCAAAATTCAGGACCGTTTCGCAGAAGGCATTTTGCTGACGCTAATCGAAG
ATGGTCCGAAAGCCCTGAAAGAGCCAGAAAACTACGATGTGCGCGCCAACGTCATG
TGGGCGGCGACTCAGGCGCTGAACGGTTTGATTGGCGCTGGCGTACCGCAGGACTG
GGCAACGCATATGCTGGGCCACGAACTGACTGCGATGCACGGTCTGGATCACGCGC
AAACACTGGCTATCGTCCTGCCTGCACTGTGGAATGAAAAACGCGATACCAAGCGC
GCTAAGCTGCTGCAATATGCTGAACGCGTCTGGAACATCACTGAAGGTTCCGATGAT
GAGCGTATTGACGCCGCGATTGCCGCAACCCGCAATTTCTTTGAGCAATTAGGCGTG
CCGACCCACCTCTCCGACTACGGTCTGGACGGCAGCTCCATCCCGGCTTTGCTGAAA
AAACTGGAAGAGCACGGCATGACCCAACTGGGCGAAAATCATGACATTACGTTGGA
TGTCAGCCGCCGTATATACGAAGCCGCCCGCTAA

Francisella tularensis nadE* **(Codon optimized for *E. coli*)**

ATGAAAATCGTTAAGGATTTTAGCCCGAAAGAATACTCCCAAAAGCTGGTAAATTG
GTTGAGCGACTCATGTATGAACTACCCGGCCGAGGGATTCGTTATCGGCTTGAGTGG
TGGTATTGACTCAGCCGTCGCGGCCTCATTGGCTGTCAAAACGGGCCTTCCTACGAC
TGCCTTAATTTTGCCGTCCGATAATAATCAGCATCAAGATATGCAGGATGCACTGGA
GTTGATCGAAATGCTTAACATTGAGCACTATACTATCTCGATCCAACCGGCGTATGA
GGCCTTCCTGGCTTCTACACAGAGTTTCACCAATCTTCAGAATAATCGTCAACTTGTC
ATTAAAGGCAACGCCCAGGCTCGTCTGCGCATGATGTATCTGTATGCATACGCCCAA
CAATACAATCGTATCGTCATTGGCACCGACAATGCGTGCGAATGGTACATGGGTTAT
TTCACGAAGTTTGGCGATGGTGCTGCCGACATTCTGCCACTGGTAAACCTTAAAAAG
TCACAAGTTTTTGAGCTGGGTAAATATCTGGACGTTCCCAAAAATATCTTAGACAAG
GCTCCGTCGGCTGGATTGTGGCAAGGGCAAACCGACGAGGATGAAATGGGGGTTAC
CTATCAAGAGATTGACGACTTCTTAGATGGGAAACAGGTTAGTGCCAAGGCCCTGG
AGCGTATCAATTTCTGGCATAACCGCTCGCATCATAAACGTAAATTAGCTTTGACCC
CAAACTTTtaa

Salmonella enterica pnuC* KA, red text indicates the KA insertion made to the DNA sequence

ATGGATTTTTTTAGTACGCACAACATACTGATTCATATTCCGATTGGCGCTGGCGGG
TACGATCTCTCGTGGATCGAAGCGGTAGGAACCATCGCCGGCCTGCTCTGTATTTGG
CTTGCCAGTCTGGAGAAGATCAGCAACTACTTTTTTGGACTGGTTAACGTTACCCTG
TTTGCGATTATTTTCTTTCAGATCCAGCTTTATGCCAGCCTGTTGCTGCAACTCTTTTT
CTTTGCCGCCAATATTTATGGCTGGTATGCGTGGTCGCGGCAAACAAAGGATAATCA
AGCCGAGCTTAAAATCCGCTGGCTGCCGTTGCCA**AAAGCA**AAAGCAATGGCATGGC
TGGCGATATGTGTGATAGCTATCGGTTTGATGACGCGATATATCGATCCCGTATTCG
CCGTCCTGACGCGCGTGGCCGTCGCCATTATGCAGATGCTGGGGTTACAGGTGACAA
TGCCCGTACTGCAACCGGACGCTTTCCCGTTCTGGGACTCTTGCATGATGGTGCTGTC
TATCGTGGCGATGATTCTGATGACACGCAAATATGTCGAAAACTGGCTCCTGTGGGT
GATAATCAACGTGATCAGTGTGGTGATTTTTGCTTTGCAGGGCGTCTATGCGATGTC
GCTGGAATATCTGATCCTGACATTTATCGCCGTGAACGGTAGCCGCCTGTGGATAAA
CAGCGCGCGGGAGCGAGGATCGCGCGCTTTCCCGTTAA

Escherichia coli* BL21 *pnuC

ATGGATTTTTTTAGTGTGCAGAATATCCTGGTACATATACCAATAGGGGCAGGCGGT
TATGATCTCTCATGGATCGAAGCGGTAGGCACGATCGCCGGGTTGCTGTGTATTGGC
CTTGCCAGTCTGGAGAAGATCAGCAACTACTTCTTTGGCCTGATCAACGTCACCTTG
TTTGGCATTATTTTCTTTCAGATTCAGCTGTATGCCAGCCTGCTATTACAGGTGTTTTT
CTTTGCCGCGAATATTTACGGTTGGTATGCGTGGTCGCGACAAACCAGTCAGAACGA
GGCGGAGTTGAAAATTCGCTGGTTGCCATTGCCGAAGGCACTCAGCTGGTTGGCGGT
TTGCGTTGTTTCGATTGGTCTGATGACGGTATTTATCAATCCGGTGTTTGCATTTTTG
ACCCGCGTGGCAGTCATGATCATGCAAGCATTAGGATTACAGGTTGTGATGCCTGAA
CTGCAACCGGACGCTTTCCCGTTCTGGGATTCATGCATGATGGTGTTATCTATCGTGG
CAATGATTCTGATGACGCGTAAGTATGTGGAAAACTGGCTGTTGTGGGTGATTATTA
ACGTGATTAGCGTCGTTATTTTTGCACTTCAGGGCGTTTACGCCATGTCTCTGGAGTA
CATCATCCTGACCTTTATTGCGCTCAACGGCAGCCGGATGTGGATCAACAGCGCACG
TGAAAGAGGCTCACGCGCGCTGTCCCATTAA

Salmonella enterica nadR

ATGCGATTTTTCCAGCAGGAGGCTCTTGTGTCATCGTTCGACTATCTCAAAACCGCG
ATTAAGCAGCAAGGTTGCACTCTGCAACAGGTGGCTGACGCCAGCGGTATGACCAA
GGGATATCTGAGTCAGTTACTTAACGCCAAAATCAAAAGCCCCAGCGCGCAAAAAC
TGGAGGCGCTACACCGTTTTCTCGGGCTGGAGTTTCCCCGCCGACAGAAAAACATTG
GCGTGGTGTTCGGTAAATTTTATCCATTGCATACCGGACACATCTACTTGATCCAGC
GCGCCTGTAGCCAGGTGGATGAGTTGCACATCATTATGGGATATGACGATACGCGC
GACCGCGGGCTGTTTGAGGATAGCGCCATGTCGCAGCAGCCCACCGTGTCGGATCG
CCTGCGCTGGTTATTGCAAACCTTCAAATACCAAAAAAATATTCGCATCCACGCCTT

TAATGAAGAGGGGATGGAGCCTTATCCGCATGGCTGGGACGTCTGGAGCAACGGCA
TTAAAGCGTTTATGGCAGAGAAGGGAATACAGCCGAGCTGGATCTACACTTCCGAA
GAGGCTGATGCGCCGCAGTATCTTGAGCATTTAGGGATTGAGACGGTGCTGGTCGAT
CCTGAACGCACGTTTATGAATATCAGTGGGGCGCAAATCCGCGAAAATCCGTTTCGT
TACTGGGAATATATTCCTACCGAAGTGAAGCCGTTTTTCGTGCGTACCGTCGCGATT
CTGGGCGGGGAATCAAGCGGCAAGTCTACGCTGGTCAATAAGCTCGCCAATATTTTT
AATACCACCAGCGCCTGGGAATATGGCCGCGACTATGTCTTTTCGCATCTGGGCGGC
GATGAGATGGCGTTACAGTATTCCGACTACGATAAAATTGCGCTGGGCCATGCGCA
ATATATTGATTTCGCAGTGAAATATGCGAATAAAGTGGCGTTTATCGATACCGATTT
CGTCACCACCCAGGCATTTTGCAAAAAATACGAAGGACGCGAGCATCCCTTTGTCCA
GGCGCTGATCGACGAGTATCGCTTCGACCTGGTGATTTTGCTGGAGAATAATACGCC
GTGGGTAGCTGACGGACTGCGAAGCCTGGGCAGTTCAGTGGATCGCAAAGCGTTCC
AGAACCTGCTGGTCGAGATGCTGAAAGAGAACAACATTGAGTTCGTTCACGTTAAA
GAGGCTGATTACGATGGTCGCTTTTTGCGCTGTGTGGAACTGGTGAAAGAGATGATG
GGCGAGCAGGGATAA

Saccharomyces cerevisiae* BY4741 *NRK1

ATGACTTCGAAAAAAGTGATATTAGTTGCATTGAGTGGATGCTCCTCCAGTGGTAAG
ACGACAATTGCGAAACTTACAGCAAGTTTATTCACGAAGGCTACATTAATTCATGAA
GATGACTTTTACAAACATGATAATGAAGTGCCAGTAGATGCTAAATATAACATTCAA
AATTGGGATTCGCCAGAAGCTCTTGATTTTAAACTTTTCGGTAAAGAATTAGATGTG
ATCAAACAAACTGGTAAAATAGCCACCAAACTTATACACAATAACAACGTAGATGA
TCCCTTTACAAAGTTCCACATTGATAGACAAGTTTGGGACGAGTTAAAGGCTAAGTA
TGACTCTATTAATGACGACAAATATGAAGTTGTAATTGTAGATGGGTTTATGATTTT
CAATAATACTGGAATATCAAAAAAATTTGATTTGAAGATATTAGTGCGTGCTCCCTA
TGAAGTACTAAAAAAAAGGAGGGCTTCCAGAAAAGGATACCAGACTTTGGATTCTT
TCTGGGTGGATCCGCCGTATTATTTCGACGAATTTGTGTATGAATCTTATCGTGCAAA
TCATGCGCAGTTATTTGTTAATGGAGACGTAGAAGGTTTACTAGACCCAAGGAAGTC
AAAGAATATAAAAGAGTTCATAAATGATGATGACACTCCAATTGCGAAACCTTTAA
GCTGGGTGTGCCAAGAGATTCTAAAGCTTTGTAAGGATTAG

***Francisella tularensis nadV* (Codon optimized for *E. coli*)**

ATGTCGTTTGATAACCTGCTGTTGATGACGGATTCCTATAAACATAGTCACCGTTAC
CAATATCCTCGCGATACCCATTATCTGCATTTTTATCTGGAATCACGCGGGACCGCT
AACAAGGATCTGGGCAACTATACGAAATTCTTCGGATTGCAGTATTACGTTAAAAAG
TATCTTTCCCAACCCATTACCCAGCAGATGATCGACGATGCAGAGAAGATCTTACTT
GCCCACGGGCTTCCGTTCTACCGTAGTGGGTTCGAGAAGATCCTTAATAATTATAAC
GGATACCTGCCGATTCGTATCCGTGCCGTGCGTGAAGGTAGTTTAATCCCGCTGCAT
AATGTATTAATGACGATTGAGTCGACGGACGAAGAGCTTTTCTGGCTTCCGGGCTTC
GTAGAAACTCTGCTGTTGAAGGTATGGTACCCAACGACTGTAGCTACGATTAGCTTT
AATATCAAACAACTGATTAAACGTTACTTGTTGGAGACGGCAGACTCGCTTGATAAG

TTAGACTTTATGTTGCATGACTTTGGATACCGCGGTGTCTCTAGTGAGGAGTCAGCA
GGTATTGGGGGGCCGCGCATCTGACCAATTTTTTGGGCACCGATACATTAGCGGCC
CTTCATGTTTGTAAAGAGTTCTATGCGGAGGACATGGCAGGATTTTCCATCCCTGCG
TCGGAACATTCAACTATGACTAGCTGGGGCGTGGGGACCGAGTGTGAGCGCGAAGC
GTTTGAAAATATGATTGCGCAGTTCGGTGACTCTTCGGTCTTATATGCTTGTGTCTCT
GACTCATGGGACTTTAAAAAAGCGATCCAGACCTGGGTAGACTTGAAAGACCGCGT
TACCGCCAAAAAGGCGAACTTAGTAATCCGTCCAGACAGTGGCGACGCCGTAGATA
ACATTTTGTACGCGCTTTATGAACTTGACAAAGGGTATGGATCACGTTTAAATAGTA
AGGGGTACAAAGTTTTAAACAATGTAGCACTTATTCAAGGGGACTCTGTTTCTATTT
CGTTAGCGAAGAAAGTTTTAGAGGCCATGAAAATTCAAGGCTACTCCGCAGAGAAC
ATTGCATTCGGGATGGGAGGGGCTCTTCTGCAAGGGAACTACGAATCGTCGATCAA
CCGCGACAGCTTCAAATTCGCAATCAAATGTTCTGCTATTATGCGCGGTAATACTTT
AATCGGCGTTAAGAAGGAGCCAATTACCGATCTTGCTAAGAAATCAAAACAGGGTC
GTTTGGATCTTATTAAGGACGCGAAAGGAAATTACAAAACGATCGTACTGGACGAC
TCGTATGCGTTAGGTGAGTATCATCCGGAATCTCAATTGCAAACCTACTATGATAAT
GGCGAGATCAAGTTTGAACAGAGCCTTGCCCAGATCCGTAATTACACAAATTAA

Ralstonia solanacearum nadV **(Codon optimized for *E. coli*)**

ATGCAGAACGACCTGCCTGGTTTGTCCGCTATCCTTAGCAACCCAATCTTAAATACC
GACAGTTACAAGGCGTCGCATTACCTGCAATACCCAGCCGGTACTTCGGCGATGTTC
TCCTACGTAGAATCCCGTGGAGGTCGTTATGATCGTACCGTTTTCTTCGGACTTCAAA
TGCTGGCAAAGGAATACTTATGCCGTCCTATTACCCCTGCTATGATCGATGCTGCCC
GCGGGTTTTTCGCAGCACACGGGGAGCCGTTTAACGAGGCGGGATGGCGTTATATTG
TTGCCCGTTATGATGGCTATCTGCCCGTACGTATCCGTGCGGTTCCCGAGGGGTCAG
TGGTACCTAATCACAACGTGCTGATGACAGTCGAATGTGACGATCCTGAAGTTTTCT
GGCTTGCGTCATATCTGGAAACTATGTTATTGCGCGTGTGGTATCCGATTACAGTTG
CGACCCAGAGCTGGCATCTGCGTCAACTTGTCCACCGCTACCTGGAGCAAACAAGT
GATGACCCAGGACAGTTGCCATTCAAGGTTCATGATTTCGGTGCTCGCGGTGTATCT
AGCGCGGAAAGTTCGGCTATTGGGGGAGCAGCTCACCTTGTGTCTTTCATGGGTAGT
GACACGGTTTTGGGTGTGGCCGCCGCAAACCTGTATTACAATGCTCAAATGGCCGCG
TTTTCTGTACCCGCGGCGGAGCACAGTACGATTACAGCCTGGGGACGTGCCGGGGA
AGCAGATGCGTATCGTAATATGTTACGCCAATTCGGTAAACCTGGTGCGATCGTGAG
TGTTGTCAGTGACAGTTATGACTTATTCGCCGCGCTTCGCCTGTGGGGAGGGGAATT
ACGCCAGGCAGTCATCGACTCTGGGGCTACGCTTGTCGTACGTCCCGATTCTGGCGA
CCCTCGCTCCATTGTTCTTCAGACAGTCCGCGCGCTTGATGCTTCATTTGGAGCAACA
GTGAACGGGAAAGGGTACCGTGTCCTGAACCACGTCCGCGTCATTCAAGGCGATGG
AATTAATGCAGCATCGATCGAGGCAATTCTTGCCGAGTTAGAGGCTGCGGGATATGC
GGCGGATAACATTGTATTCGGGATGGGAGGTGCCCTGTTACAACAATTAAACCGCG
ACACACAGCGCTTTGCAATGAAGTGCTCAGCAGTCCGTGTTGACGGGGCGTGGCGT
GAAGTCTGTAAAGACCCGGTGACCGACGCGGGGAAACGTTCTAAGAAAGGACGTCT
TACACTTTTGCGCAACCGTGTGAGCGGGGAGTACGCCACAGCCACTTTGCCCTTGGC

CTGGGATGATCGCCGCATCGAGGGGGAATGGGAGGATGCTCTGGTGACGGTATTCG
AGAATGGGCGTCTTTTACAGGATGTCAGCCTTGACGCGGTCCGCGCGCGCGCTCAAG
CCCATGAGTTGGCACCCGCCCTTGTCGACTGA

Synechocystis sp.* PCC 6803 *nadV

ATGAATACTAATCTCATTCTGGATGTGGACTCCTATAAAGTGAGCCACTGGTTGCAG
TATCCTCCTGACACAACGGCAATGTATTCCTATGTGGAAAGTCGTGGGGGAAGGTAT
CCTGTCACTGTCTTTTTTGGTCTCCAATACATTTTAAAGCGGTATCTGACTCAATCCA
TTGAACCCTGGATGGTGGAGGAAGCTAATCGCCTTTTGACAGCCCATGGCTTACCTT
TCAACTATGGCGGTTGGCGATACATTGCGGAGGATTTGCAGGGTCGTTTACCTGTAC
GTATTAAGGCGGTTCCAGAGGGCTCGGTCATCCCGGTTCATAATGTTTTGATGACAG
TGGAATCCACGGACCCAAAGGTTTTTTGGTTAGTTTCCTGGTTAGAAACTTTGTTGAT
GCGGGTTTGGTATCCCATTACGGTGGCAACCCAGAGTTGGCATTTAAAACAACGCAT
CTATCAATCCCTATGCCGTACTGCGGATGATCCTGATGGTGAAATCAATTTTAAACT
CCACGATTTTGGGGCCCGGGGGGTTTCTAGTGGTGAATCGTCCGGCATTGGCGGACT
GGCTCACTTAGTTAATTTCCAAGGTTCTGACACAGTAAAGGCCCTGGTGTATGGGCA
GCAATATTACAACTGCCCCATGGCGGCCTATTCGATTCCCGCCGCAGAACATTCCAC
CATTACAGCTTGGGGAAGGGAAGGGGAAGTTTTGGCCTATGAAAATATGTTGACCC
AGTTTGCCAAGCCAGGGTCGGTGTTGGCGGTGGTTTCCGATTCCTATGATCTCTGGA
ATGCCATTGACCATCTCTGGGGCGATCACCTAAGGGCACAGGTGCTTGATTCGGGGG
CTACGGTGGTTATCCGTCCGGATTCAGGTGACCCGGTGGCCATTGTGGCCCAAACTT
TGGAACGGTTGGAGGCTTGTTTTGGCAGCACCCTCAACAGTAAGGGCTTTCGAGTTC
TAAATGCTGTGCGGGTTATCCAAGGGGATGGGGTTGATGAAGAGAGTATCAGCGCC
ATTCTAGAGAAGACTGAGAGCCTTGGCTTTAGTACTACTAATTTAGCTTTTGGTATG
GGGGGAGCTTTGTTGCAAAAGGTGAATCGGGATACCCAAAAATTTGCCATGAAGTG
CAGTGAGGTAACGGTGGAGGACAAGGCGATCCCTGTTTATAAAGACCCTGTTACTG
ATCCTGGTAAAACTAGCAAAAAGGGGCGATTATCCCTGGTTAAAACTGACTCTGGTT
ATGGCACTGTACCCACTTCTTCTGAGGATTTATTGCAGGTTGTCTATGAAAATGGAC
ATTTACTGCAAGACCAATGCTTGGATGCTATTCGTCAACGAGCCTGGCCATTAATCA
GGGTCAATGTTCCCGCAAGCTAG

Synechococcus elongatus* PCC 7942 *nadV

ATGGACCTCAATCTTCTGTTCGATACCGACTCATACAAAGTCAGCCACTGGCTGCAA
TATCCTGCCGATACGACTGCGATCGGAGCTTATTTAGAAAGCCGGGGTGGAGATTGC
TCGCACACGCTCTTTTTTGGCTTGCAATATCTACTACTGCGTTATTTCTTCCAGCCAA
TCACTAGCGCTGACATTCAAGAAGCCGCCGCGCTGTTTCAAGCGCATGGGCTGCCTT
TCAATCAAGCGGGCTGGCAACGAGTTTGCGATCGCTATGGCGGGTATTTACCTTTAC
GAATTCGGGCTGTCCCGGAAGGTAGCCTTGTCCCCACCGGCAATATCTTGCTGACAG
TGGAATCGACCGATCCTGAATTGGCTTGGCTGGCCACTTGGGTTGAGACACTACTGC
TGCGGGTTTGGTATCCGATAACTGTGGCTACACGCAGTTGGCAGCTTCGGCAAATCA

TTCAGCAAGCGCTGGAGCAATCAGCCGAAAATCCAGCAGCTGAAATTGACTTCAAA
CTGCATGACTTTGGATCACGCGGGGTATCGAGCCAAGAAAGTGCTGCAATCGGCGG
GCTGGCTCATTTGGTCAACTTTCAAGGCACTGATACGATCGCTGCGTTACTGGCAGG
ACAGCGCTATTACGATTGCGCGATCGCTGGCTTTTCGATTCCGGCGGCGGAGCATTC
AACGATTACGGCTTGGGGCCCATCGGGTGAGTTAGATGCTTACCGCAATATGCTCGA
TCGCTTTGCAAATCCGGGATCTGTGGTGGCTGTTGTATCGGACTCCTATGATCTCTGG
CATGCCGTCGATCAGCTTTGGGGTGAGGATCTCCGCGATCGCATTTTGCAATCGGGA
GCAACCGTTGTCATTCGGCCTGACTCAGGCAATCCTGAGCAGATTGTGCCGGAATTA
CTGCGTCGTTTGGCCGCTAAGTTCGGCTGCGATCGCAATCAGAAGGGTTATCAAGTT
TTGCGATCGGTGCGGGTGATTCAGGGCGATGGGATCACAGTGGACAGTCTGCCCAA
AGTTCTGCAAGCGGTTATGGCCGCTGGCTTTAGTGCCAGTAATGTCGCTTTTGGCAT
GGGTGGCGGGCTGTTGCAGCAGGTCAATCGCGATACCCAACGCTTTGCCTACAAGTG
CAGCTGGATCGAGCGATCGGGACAAGTGATTCCCATTTGCAAGCGACCAGCCACGG
ATCTGCGCAAGGCTAGCAAAGCAGGACGCTTGGATTTAATTCGCGATCGCGAGGGG
CAATACCGAACAGTCTCGTTACTGACGTCAGAGCCTGACCCGCAATCCTGCCTGCAA
ACGGTGTTTGAAAATGGTGCGATCGTGCGGCGACAAAGCTTGCAGGAAATCCGCGA
TCGCGCTCGTTCTGAGACACGCTAG

***Haemophilus ducreyi nadV* (Codon optimized for *E. coli*)**

ATGGATAATTTATTAAATTACTCTTCGCGTGCTTCGGCCATTCCGTCGCTGTTGTGTG
ACTTTTATAAGACATCGCACCGCATTATGTATCCCGAGTGTAGTCAAATCATTTACTC
GACCTTCACACCACGTTCCAATGAACAAGCTCCATACCTGACACAAGTTGTCTCATT
CGGCTTTCAGGCATTTATTATTAAGTACCTTATTCATTACTTCAACGACAATTTTTTC
TCACGCGATAAACATGATGTAGTTACTGAATACTCGGCCTTTATCGAGAAGACTCTT
CAATTAGAAGATACCGGAGAGCATATCGCGAAGCTGCACGAATTGGGCTACCTTCC
GATTCGTATCAAGGCAATCCCCGAGGGGAAAACCGTGGCAATCAAGGTCCCTGTAA
TGACCATCGAGAATACCCATAGTGACTTCTTTTGGTTAACCAATTACTTGGAAACAT
TGATCAACGTTTCGTTGTGGCAGCCGATGACCTCTGCCTCGATTGCATTCGCATATCG
TACTGCCCTTATCAAGTTTGCGAATGAAACGTGCGATAATCAAGAGCATGTCCCCTT
TCAGAGCCACGATTTTTCTATGCGTGGAATGAGTTCCCTGGAATCAGCCGAAACATC
TGGTGCTGGACACTTAACTTCGTTCCTGGGTACGGACACCATTCCGGCGCTTTCCTTT
GTCGAGGCTTATTACGGCAGTAGTAGCCTTATTGGTACATCGATCCCTGCAAGTGAA
CATTCTGTGATGTCCTCACATGGAGTTGACGAGTTAAGTACTTTCCGCTACTTAATGG
CTAAGTTCCCGCATAACATGCTGAGTATCGTGAGTGATACGACTGACTTTTGGCACA
ATATCACGGTCAACCTTCCGTTATTGAAGCAAGAAATCATTGCGCGTCCAGAGAATG
CTCGTCTGGTTATCCGCCCTGATTCAGGGAACTTCTTTGCAATCATCTGCGGAGACCC
AACCGCGGACACCGAACACGAACGCAAGGGTCTTATCGAATGTTTGTGGGACATCT
TCGGAGGGACTGTCAACCAAAAGGGTTATAAAGTTATCAATCCACACATCGGAGCG
ATCTATGGGGACGGCGTAACCTATGAAAAGATGTTCAAGATCTTGGAAGGTCTTCAA
GCCAAAGGATTTGCCTCCAGCAACATTGTATTTGGCGTGGGGGCGCAGACTTACCAG
CGTAATACTCGCGATACATTGGGATTTGCACTGAAGGCCACCTCTATTACTATTAAT

GGAGAGGAGAAGGCAATTTTCAAGAATCCCAAAACAGATGATGGCTTTAAGAAGAG
CCAGAAGGGACGCGTCAAAGTTCTTTCCCGCGATACTTACGTAGATGGCTTAACAAG
CGCTGACGACTTTAGCGACGACCTTCTGGAGCTGCTTTTCGAGGACGGCAAGCTTTT
ACGCCAGACCGATTTCGACGAAATTCGTCAAAATCTGCTTGTGTCCCGTACCACTTT
GTAA

References

[1] Stancek M, Isaksson LA, Rydén-Aulin M. fusB is an Allele of nadD, Encoding Nicotinate Mononucleotide Andeylyltransferase in *Escherichia coli*. Microbiology. 2003;149(9):2427-33.
